# Combined effect of arginine and fluoride on the growth of *Lactobacillus rhamnosus GG*

**DOI:** 10.1038/s41598-020-79684-2

**Published:** 2021-01-13

**Authors:** Mohammed Nadeem Bijle, Manikandan Ekambaram, Edward C. M. Lo, Cynthia Kar Yung Yiu

**Affiliations:** 1 Paediatric Dentistry, Faculty of Dentistry, The University of Hong Kong, Hong Kong, Hong Kong SAR; 2 Paediatric Dentistry, Faculty of Dentistry, University of Otago, Dunedin, New Zealand; 3 Dental Public Health, Faculty of Dentistry, The University of Hong Kong, Hong Kong, Hong Kong SAR

**Keywords:** Microbiology techniques, Drug development

## Abstract

The objectives of the in vitro study were: (1) to investigate the effect of combining L-arginine (Arg) and NaF on the growth of *Lactobacillus rhamnosus *GG (LRG); and (2) to identify an optimum synergistic concentration for the synbiotic (Arg + LRG)-fluoride (SF) therapy. 1% Arg + 2000-ppm NaF (A-SF) and 2% Arg + 2000-ppm NaF (B-SF) demonstrated antagonism against LRG (FIC > 4.0). Both XTT (2,3-bis-(2-methoxy-4-nitro-5-sulfophenyl)-2H-tetrazolium-5-carboxanilide) and WST-8 (2-(2-methoxy-4-nitrophenyl)-3-(4-nitrophenyl)-5-(2,4-disulfophenyl)-2H-tetrazolium, monosodium salt) assays showed that A-SF and B-SF enhanced the growth of LRG when compared to 2000-ppm NaF and LRG control. Colony forming units, bacterial weight, and biofilm thickness of A-SF and B-SF were significantly higher than 2000-ppm NaF and LRG control. Biofilm imaging depicted that 2000-ppm NaF inhibited biofilm formation; while 1%/2% Arg, A-SF, and B-SF increased biofilm growth of LRG. Lactic acid formation was the lowest for 2000-ppm NaF, followed by A-SF and then B-SF. The SF buffer potential after 24 h was the highest for B-SF, and then A-SF. Biofilm pH for B-SF was closest to neutral. Fluoride, Arg and LRG bioavailability remained unaffected in B-SF. The relative gene expression for *arcA*, *argG*, and *argH* was significantly higher for B-SF than the respective controls. In conclusion, combining 2% Arg, 2000-ppm NaF, and LRG provides an optimum synbiotic-fluoride synergism.

## Introduction

A homeostatic host-microbe interaction is imperative to effectively maintain or restore health^1^. Microbial host colonization forms surface biofilms that determines health and disease. Dominance of bacterial pathogens in the biofilms leads to ecological dysbiosis, thereby contributing to infectious diseases. Persistence of dysbiosis causes chronic diseases like dental caries and periodontal diseases in microbial dense and diverse human anatomical locations like oral cavity. Dysbiosis-associated pathogenic microbial communities are embedded in extra-cellular matrix that limits the anti-microbial effect of antibiotics by retarding drug diffusion eventually resulting in anti-microbial resistance. Indiscriminate antibiotic use has further contributed to chronic biofilm dysbiosis that proliferates harmful pathogenic manifestations^2,3^. Nevertheless, biofilms play an integral role in health and thus, eradicating biofilms would limit health-associated beneficial effects. Therefore, biofilm microbiome modulation to restore ecological homeostasis is a promising approach for prevention of dysbiosis-related diseases.

Biofilm modulators like prebiotics and probiotics target to shift biofilms from dysbiosis to microbiome symbiosis, thereby restoring homeostasis. Prebiotics are dietary additives that optimize the metabolism of certain oral bacteria and enhance the growth of pro-healthy bacteria like oral commensals. Probiotics are living supplementary organisms that provide beneficial health effects by replenishing healthy microbial flora. A combination of pre- and probiotics mutually imparting beneficial effects termed as synbiotics allows for a prevailing delivery of probiotics^4^. Recently, pre- and probiotics have been combined to develop novel synbiotics for suppressing oral pathogens^5^. Although the preventive potential of synbiotics for general health is recognized, its effect on oral health is yet to be discerned projecting similar effects as identified with systemic synbiotics usage^4,5^.

The framework of oral dysbiosis is disease-dependent as caries affects the hard tissues of teeth while periodontal diseases affect its supporting structures. Dental caries is an endogenous biofilm-mediated disease caused by pathogenic ecological shift by acidogenic/aciduric bacteria (e.g. *Streptococcus mutans*), creating acidic microenvironments and resulting in demineralization of hard tissues^6^. With periodontitis, the oral microbiome shifts from gram-positive aerobes to gram-negative anaerobes^7^. Probiotic supplementation aids to sustain homeostasis in the oral cavity; however, its effect depends a lot on the type and viability of probiotic bacteria delivered^8–13^.

Dental caries control with fluorides (F) is evident with the decline in caries prevalence globally^14^. However, it has been estimated that 2.4 billion people suffer from caries of permanent teeth and 486 million children suffer from caries in primary teeth^15^. Fluoride inhibits tooth demineralization under acidic conditions and enhances remineralization in neutral conditions, thereby reducing net mineral loss. Despite this, F has limited action on oral biofilms and may not be effective in complete caries prevention as the disease occurs sequalae to supra-gingival biofilm dysbiosis^16^. Therefore, supplementing F with other preventive measures that modulate oral microbiome and restore ecological homeostasis is the key to caries prevention.

Arginine (Arg), a prebiotic biofilm modifier, is metabolized by arginolytic oral commensals (e.g. *Streptococcus sanguinis*, *Streptococcus parasanguinis*, and *Streptococcus gordonii*) to produce ammonia by arginine deiminase system (ADS). The generated ammonia modulates oral pH, favours the growth of health-associated oral commensals and suppresses mineral loss from tooth substrates^17^. Arginine has numerous health benefits with minimal risks, ranging from alleviating hypertension, metabolic disorders, GI disorders and erectile dysfunction^18,19^. It inhibits the growth of cariogenic bacteria *S. mutans*, impacts biofilm matrix by reducing biofilm biomass with water-insoluble EPS production in extracellular matrix, and destabilizes mature oral biofilms^20–23^. It has been shown that the combination of Arg with F synergistically reduce *S. mutans* and enrich *S. sanguinis* within multi-species biofilms^24^. Arginine has been incorporated in commercial F-containing toothpastes to provide enhanced protection against caries for high-risk patients, whereby several clinical trials have indicated a superior caries-preventive effect of the Arg and fluoride toothpastes compared to the control toothpaste with fluoride alone^25–34^. However, long-term use of Arg poses a risk of increased plaque alkalization and overgrowth of oral anaerobes such as *Porphyromonas gingivalis*^24,35^. Thus, strategies are needed to channelize consumption of Arg to limit contributory plaque overalkalization.

*Lactobacillus rhamnosus* GG (LRG) is the world’s most researched probiotics, demonstrating profound systemic benefits. Although the viability of LRG in the gastric and oral environments is higher compared to other probiotics^36^, the effective delivery of viable functional bacteria against harsh conditions is challenging. Thus, delivery systems that enhances the probiotic viability are much needed. The antagonistic effect of LRG against cariogenic bacteria (*S. mutans*) and periodontal pathogen (*P. gingivalis*) is evident through clinical trials^8,11,12^ but the effect of LRG in the oral cavity is transient as it does not achieve long-standing colonization in the oral biofilms. Recently, prebiotics has been combined with probiotics to develop synbiotics with complementary actions that prolong the beneficial effects of probiotics, enhance the growth and survival of health-associated oral microbiota.

The mechanism of action of fluoride may be enhanced with the recently developed synbiotics. The combination will provide an enduring ecological-based caries-preventive strategy with two-fold synergistic anti-caries effect, firstly targeting biofilm control by synbiotics and secondly enamel remineralization by fluoride. The synergistic effect of Arg and F has been shown in previous studies^24–34,37^. However, the effect of synbiotics (Arg + LRG) in the presence of F remains undiscovered as F could impart its antimicrobial properties limiting the delivery of viable probiotic bacteria as seen with planktonic cariogenic bacteria (*S. mutans*)^38^. Thus, the objective of the study were: 1) to investigate the effect of combining Arg and NaF on the growth of *Lactobacillus rhamnosus *GG; and 2) to identify an optimum synergistic concentration for the synbiotic (Arg + LRG)-fluoride (SF) therapy targeted for oral ecological homeostasis. The null hypothesis tested in the present study was that the combination of Arg and NaF has no effect on the growth of LRG.

## Results

### Fractional inhibitory concentration

**Figure 1:**
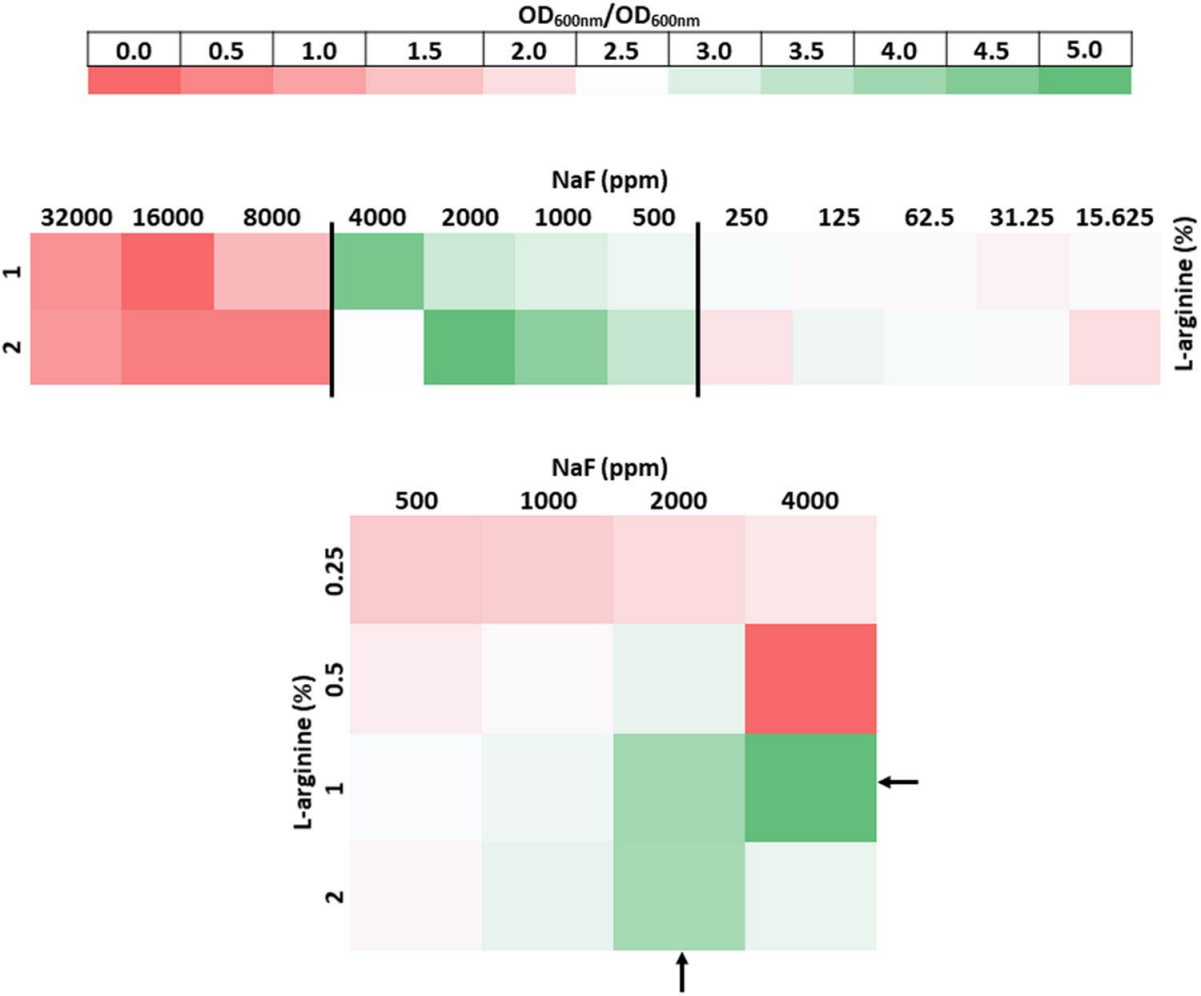
Checkerboard microdilution assay (CMA) showing the effect of Arg and NaF on the growth of LRG. The boundaries with black straight lines indicate the concentrations of Arg/NaF that augment the growth of LRG. The black arrows indicate the concentrations of Arg/NaF outlined for further experimental analysis. The representation is based on the minimum inhibitory concentrations (MICs) and computed fractional inhibitory concentrations (FICs) Index (ƩFIC) with CMA.

The higher NaF concentration (> 4000 ppm) with 1%/2% Arg had a ƩFIC < 1.0, indicating indifference against LRG. At the other end, NaF concentration < 500 ppm with 1%/2% Arg exhibited indifferent effect on LRG growth (ƩFIC > 1.5). The ƩFIC ≥ 2.0 were observed for 500–4000 ppm NaF with 1%/2% Arg against LRG. Whereas, the FIC index for 1% Arg + 2000 ppm NaF, 2% Arg + 2000 ppm NaF and 1% Arg + 4000 ppm NaF against LRG were 4.44, 4.36 and 5.72, respectively (Fig. 1).

Thus, 1% Arg + 2000-ppm NaF (A-SF), 2% Arg + 2000-ppm NaF (B-SF), and 1% Arg + 4000-ppm NaF demonstrated significant antagonism against LRG growth (ƩFIC > 4.0), indicating that the outlined concentrations of NaF + Arg enhanced the growth of LRG.

### Probiotic cell metabolic activity, viability, vitality, and biofilm thickness

#### Probiotic cell metabolic activity

**Figure 2:**
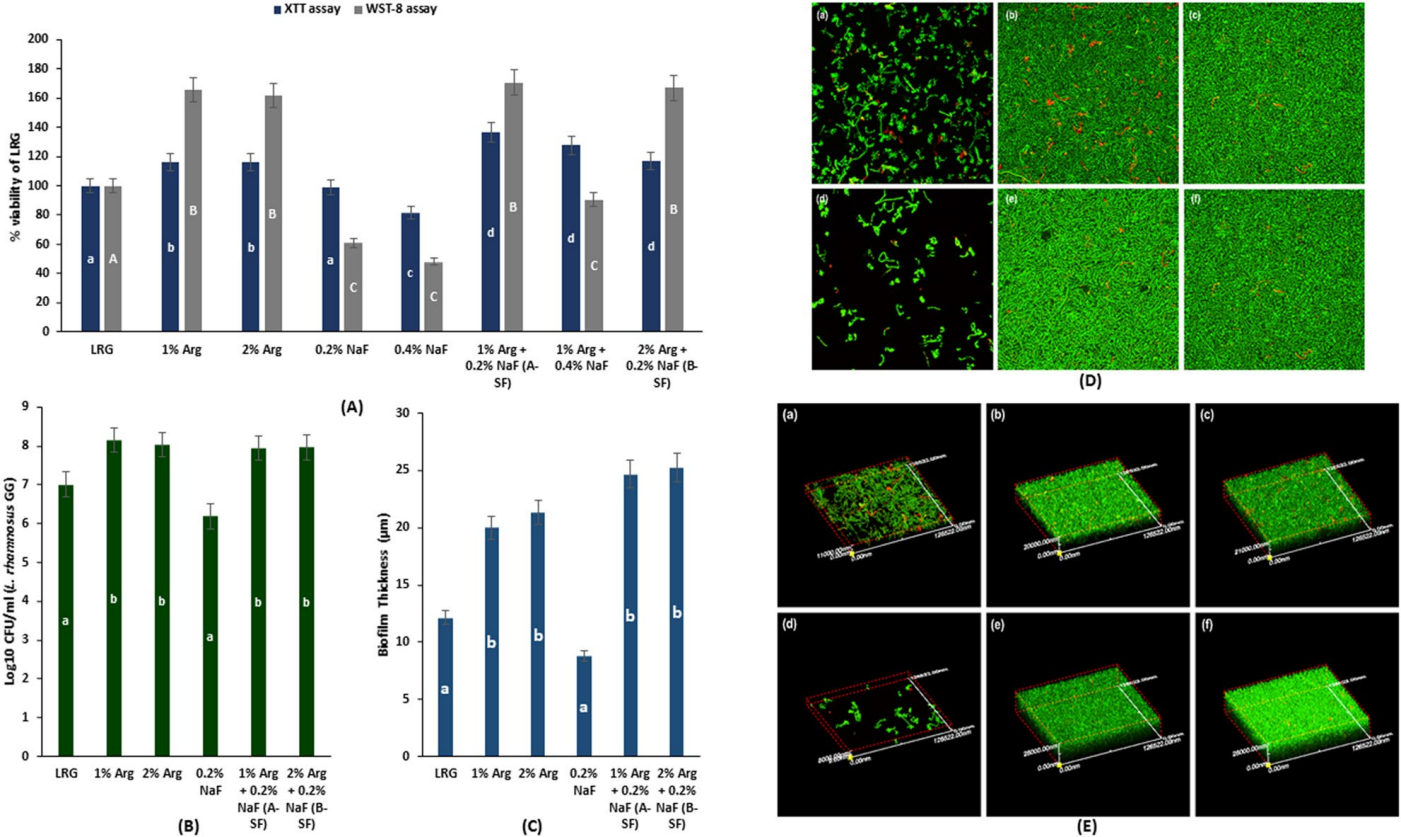
*Lactobacillus rhamnosus* GG cell metabolic activity, viability, vitality, and biofilm thickness: (**A**) LRG cell metabolic activity assessed using XTT and WST-8 assay eventually measured as %viability of LRG treated with A-SF, B-SF, 1% Arg + 0.4% NaF, and respective intervention controls. The data was normalized to LRG control. (**B**) The LRG cell viability assessed by colony forming units for A-SF, B-SF, and the respective controls. (**C**) The biofilm thickness determined using confocal laser scanning microscopy (in µm) for A-SF, B-SF, and the respective controls. (**D**) 2-D confocal laser scanning microscopy images (×100) for LRG treated with A-SF, B-SF, and respective controls: (**a**) LRG; (**b**) 1% Arg; (**c**) 2% Arg; (**d**) 0.2% NaF; (**e**) 1% Arg + 0.2% NaF; and (**f**) 2% Arg + 0.2% NaF. (**E**) 3-D scaled confocal laser scanning microscopy images (×100) for LRG treated with A-SF, B-SF, and respective controls: (**a**) LRG; (**b**) 1% Arg; (**c**) 2% Arg; (**d**) 0.2% NaF; (**e**) 1% Arg + 0.2% NaF; and (**f**) 2% Arg + 0.2% NaF. Both 2-D and 3-D confocal imaging demonstrate the enhanced cell density and LRG vitality with A-SF, B-SF, 1% Arg, and 2% Arg. Different lowercase (**a**–**d**)/uppercase (**A**–**C**) English alphabets indicate significant differences between treatment groups at p < 0.05.

The results of XTT (2,3-bis-(2-methoxy-4-nitro-5-sulfophenyl)-2H-tetrazolium-5-carboxanilide) assay showed that A-SF, 1% Arg + 4000-ppm NaF, and B-SF significantly enhanced the metabolic activity of LRG compared to 1%/2% Arg, 0.2%/0.4% NaF, and the LRG control (*p* < 0.05) (Fig. 2A). No significant difference in metabolic activity of LRG was found among A-SF, 1% Arg + 4000-ppm NaF, and B-SF (*p* > 0.05).

Conversely, a high-sensitivity WST-8 (2-(2-methoxy-4-nitrophenyl)-3-(4-nitrophenyl)-5-(2,4-disulfophenyl)-2H-tetrazolium, monosodium salt) assay demonstrated that A-SF, B-SF, and 1%/2% Arg had a significantly higher LRG metabolic activity than 1% Arg + 4000-ppm, 0.2%/0.4% NaF, and the LRG control (*p* < 0.05) (Fig. 2A). No significant differences in LRG metabolic activity were observed among A-SF, B-SF, and 1%/2% Arg.

The results of the probiotic cell metabolic activity with XTT and WST-8 assays indicated that A-SF and B-SF significantly enhanced the growth of LRG when compared to 0.2%/0.4% NaF and LRG control (*p* < 0.001); whereas 0.4% NaF alone significantly inhibited the growth of LRG showing an antimicrobial effect against LRG.

#### Probiotic cell viability

The LRG viability of A-SF, B-SF, and 1%/2% Arg groups, estimated by CFU on agar plates, was significantly higher than that of the LRG control and 0.2% NaF (*p* < 0.001) (Fig. 2B). No significant differences in LRG viability were found among the A-SF, B-SF, 1%/2% Arg groups (*p* > 0.05). Thus, the results of CFU confirmed outcomes of WST-8 assay, indicating that 1%/2% Arg, A-SF and B-SF significantly enhanced the growth of LRG compared to the LRG control (*p* < 0.001).

### Biofilm imaging

#### Confocal laser scanning microscopy

The biofilm thickness data derived using 3-D scanned confocal images are shown in Fig. 2C. The 2-D and 3-D biofilm scanned confocal images of LRG treated with A-SF, B-SF and the respective controls are shown in Fig. 2D,E.

Thickness of the biofilm in the A-SF, B-SF, and 1%/2% Arg groups, measured in µm, was significantly higher than that of LRG treated with 0.2% NaF and the LRG control (*p* < 0.001) (Fig. 2C). No significant difference in biofilm thickness was observed among A-SF, B-SF, and 1%/2% Arg (*p* > 0.05) (Fig. 2C). The biofilm thickness results indicated that A-SF and B-SF enhanced the growth of LRG to form a biofilm, thereby improving LRG sustainability.

Fewer live cells (stained green) were seen in the LRG control (Fig. 2D(a),E(a)) and in LRG treated with 0.2% NaF (Fig. 2D(d),E(d)); whereas the LRG treated with 1% (Fig. 2D(b),E(b))/2% Arg (Fig. 2D(c),E(c)) and A-SF (Fig. 2D(e),E(e))/B-SF (Fig. 2D(f),E(f)) showed comparatively higher live cell density and biofilm thickness (Fig. 2C–E).

#### Scanning electron microscopy

**Figure 3:**
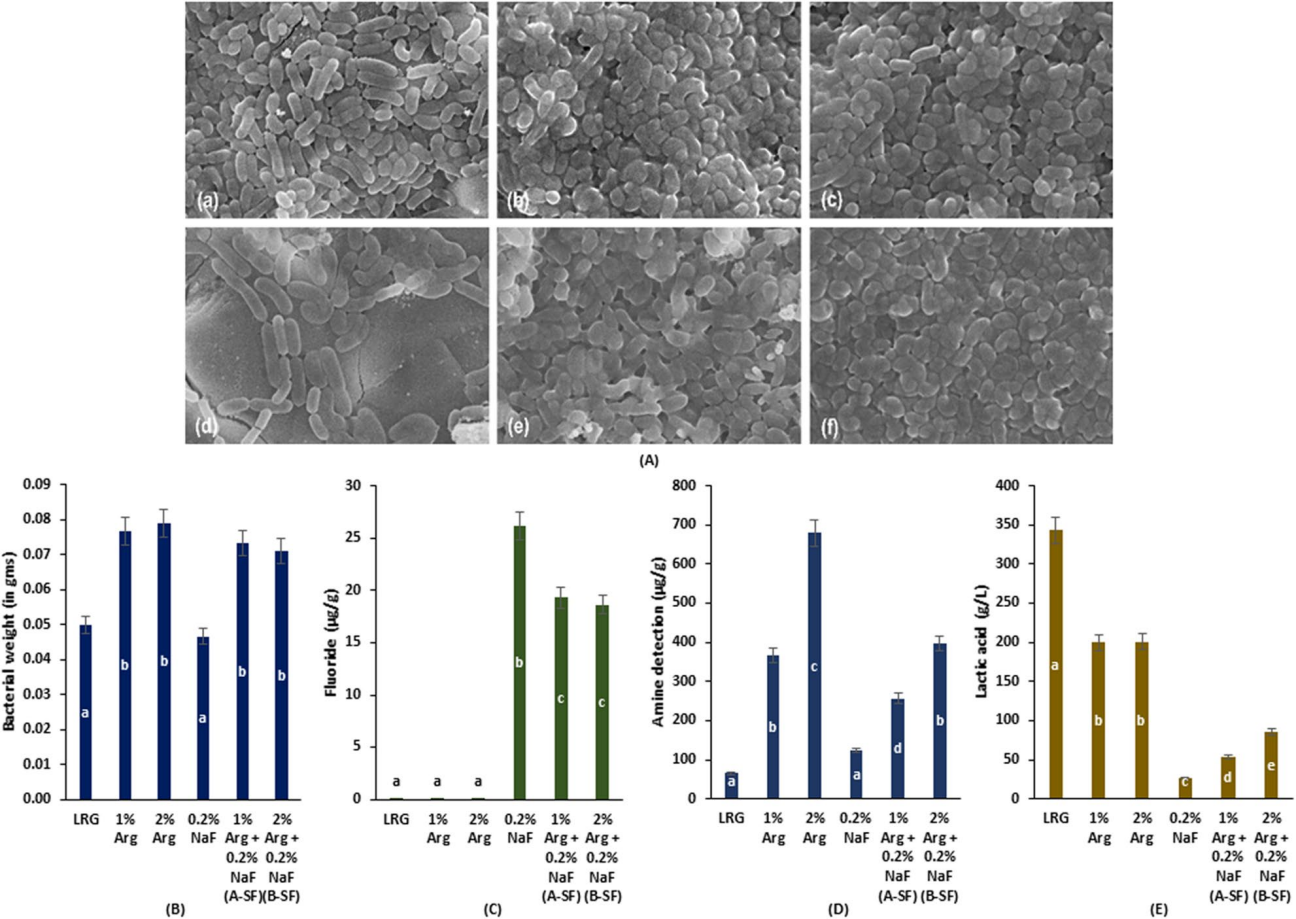
(**A**) Scanning Electron Microscopy Biofilm Imaging (×8000, 15 kV) for LRG treated with A-SF, B-SF, and respective controls: (**a**) LRG; (**b**) 1% Arg; (**c**) 2% Arg; (**d**) 0.2% NaF; (**e**) 1% Arg + 0.2% NaF; and (**f**) 2% Arg + 0.2% NaF. (**B**) Bacterial weight (in gms) determined for LRG treated with A-SF, B-SF, and respective controls. (**C**) Biofilm F content (in µg/g) determined for LRG treated with A-SF, B-SF, and respective controls. (**D**) Biofilm amine content (in µg/g) determined for LRG treated with A-SF, B-SF, and respective controls. (**E**) Lactic acid generated in the spent media after 24 h treatment of LRG with A-SF, B-SF, and respective controls. Different lowercase (a–e) English alphabets indicate significant differences between treatment groups at *p* < 0.05.

The biofilm SEM images are presented in Fig. 3A. The SEM imaging confirmed that 0.2% NaF (Fig. 3A(d)) inhibited LRG growth and biofilm formation as compared to the other groups. The biofilm architecture of A-SF (Fig. 3A(e)) and B-SF (Fig. 3A(f)) showed populous LRG cells similar to 1% Arg (Fig. 3A(b)) and 2% Arg (Fig. 3A(c)). A distinctive cluster of discrete LRG cells could be identified in the LRG control (Fig. 3A (a)) as opposed to A-SF/B-SF treated LRG group. Thus, the SEM imaging results are in conformity with confocal imaging, showing the formation of highly organised LRG biofilm when treated with A-SF or B-SF, whereas NaF inhibited the biofilm formation of LRG.

### Bacterial weight, biofilm fluoride and amine content, and generated lactic acid

The determined bacterial weight in the treated LRG biofilms is shown in Fig. 3B. Similar to the results of biofilm imaging, the bacterial weight of the A-SF/B-SF and 1%/2% Arg treated LRG was significantly higher than that of the LRG control and LRG treated with 0.2% NaF (*p* < 0.001), thereby confirming that A-SF/B-SF augmented the growth of LRG.

The biofilm F content with 0.2% NaF was significantly higher than the other groups (*p* < 0.001) (Fig. 3C). The biofilm F content of the A-SF/B-SF treated LRG was significantly lower than that of 0.2% NaF treated LRG (*p* < 0.05). No F content was determined in the LRG control and 1%/2% Arg treated LRG biofilms. The results of biofilm F content showed that A-SF/B-SF treatment of LRG significantly reduced the free F availability in the biofilm compared to 0.2% NaF (*p* < 0.05). The detected amine content indicated that the amount of biofilm amino acids in the 2% Arg group was significantly higher than the other groups (*p* < 0.001); whereas the amine content in the 1% Arg and B-SF groups was lower than the 2% Arg but higher than the A-SF, LRG control and 0.2% NaF treated LRG groups (*p* < 0.05) (Fig. 3D). Similar to the results of biofilm F content, the free amine content in the biofilm (indicating Arg availability) was significantly decreased in the presence of NaF with A-SF/B-SF treatment.

The amount of lactic acid generated in the study groups is shown in Fig. 3E. The least amount of lactic acid was found in 0.2% NaF whereas the highest amount was found in the LRG control (*p* < 0.001). A-SF and B-SF-treated LRG had significantly higher lactic acid than 0.2% NaF, but lower than 1%/2% Arg (*p* < 0.05). The lactic acid in the spent media of B-SF was significantly higher than A-SF (*p* < 0.05). Thus, lactic acid production was significantly inhibited with the SF therapy.

Overall, the biofilm chemical parameters like biofilm F/amine content and generated lactic acid were significantly reduced by A-SF/B-SF therapy, despite augmented growth of LRG.

### pH of spent media, buffer capacity of added buffers, biofilm pH

**Figure 4:**
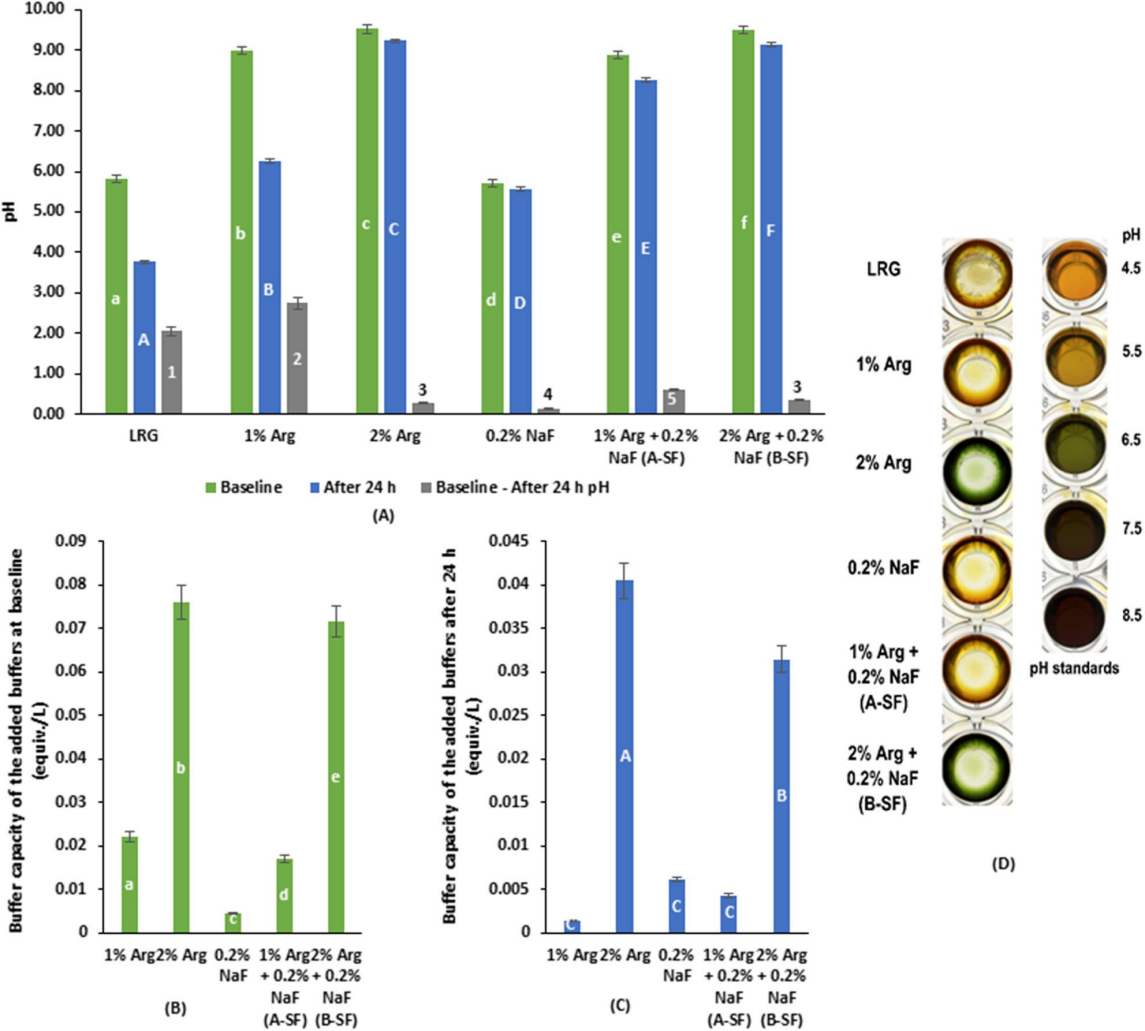
(**A**) Baseline and after 24 h pH of the spent media in the treatment of LRG with A-SF, B-SF, and respective controls. (**B**) The buffer capacity of added buffers (Arg/NaF) for the treatment of LRG with A-SF, B-SF, and respective controls at baseline immediately after treatment. (**C**) The buffer capacity of added buffers (Arg/NaF) for the treatment of LRG with A-SF, B-SF, and respective controls after 24 h of treatment. (**D**) The biofilm pH colorimetric indicator assay performed in a 96-well microplate for LRG treated with A-SF, B-SF, and respective controls after 24 h of LRG growth to biofilm against HEPES buffer pH standards treated with pH indicator as a reference for qualitative assessment. Different lowercase (a–f)/uppercase (A–F) English alphabets and numbers (1–5) indicate significant differences between treatment groups at *p* < 0.05.

The spent media pH measured at baseline (immediately after treatment) was the lowest in 0.2% NaF; while the highest was in 2% Arg (p < 0.001). The pH at baseline for B-SF was significantly lower than 2% Arg but higher than 1% Arg > A-SF > LRG control (*p* < 0.05) (Fig. 4A). The pH at 24 h for 2% Arg remained the highest followed by B-SF > A-SF > 1% Arg > 0.2% NaF > LRG control (*p* < 0.05) (Fig. 4A). The baseline pH of the spent media was significantly higher than the 24 h pH for all the groups (p < 0.001), with significantly higher difference between baseline and 24 h pH seen in 1% Arg > LRG > A-SF > 2% Arg = B-SF > 0.2% NaF (*p* < 0.05) (Fig. 4A).

The buffer capacity of Arg/NaF at baseline was the highest for 2% Arg, followed by B-SF > 1% Arg > A-SF > 0.2% NaF (*p* < 0.001) (Fig. 4B). At 24 h, the buffer capacity of Arg/NaF was the highest for 2% Arg, followed by B-SF > 0.2% NaF = A-SF = 1% Arg (*p* < 0.05) (Fig. 4C). The results of the spent media pH and the buffer potential of Arg/NaF indicated that 2% Arg and B-SF had a significantly higher potential to resist pH change than the other groups. Furthermore, the results showed that both 2% Arg and B-SF aid to maintain the media pH around neutral.

The qualitative assessment of biofilm pH showed that 1% Arg, 0.2% NaF, A-SF, and LRG control had a pH close to 4.50; whereas the pH for 2% Arg and B-SF was closest to neutral (Fig. 4D). Thus, the pH colorimetric indicator assay results are in concordance with the assessment results of the buffer capacity of Arg/NaF, thereby demonstrating that B-SF is a promising SF therapy that assists maintaining pH close to neutral irrespective of the enhanced growth of lactic acid bacteria LRG.

### Synbiotic-fluoride therapy bioavailability

**Figure 5:**
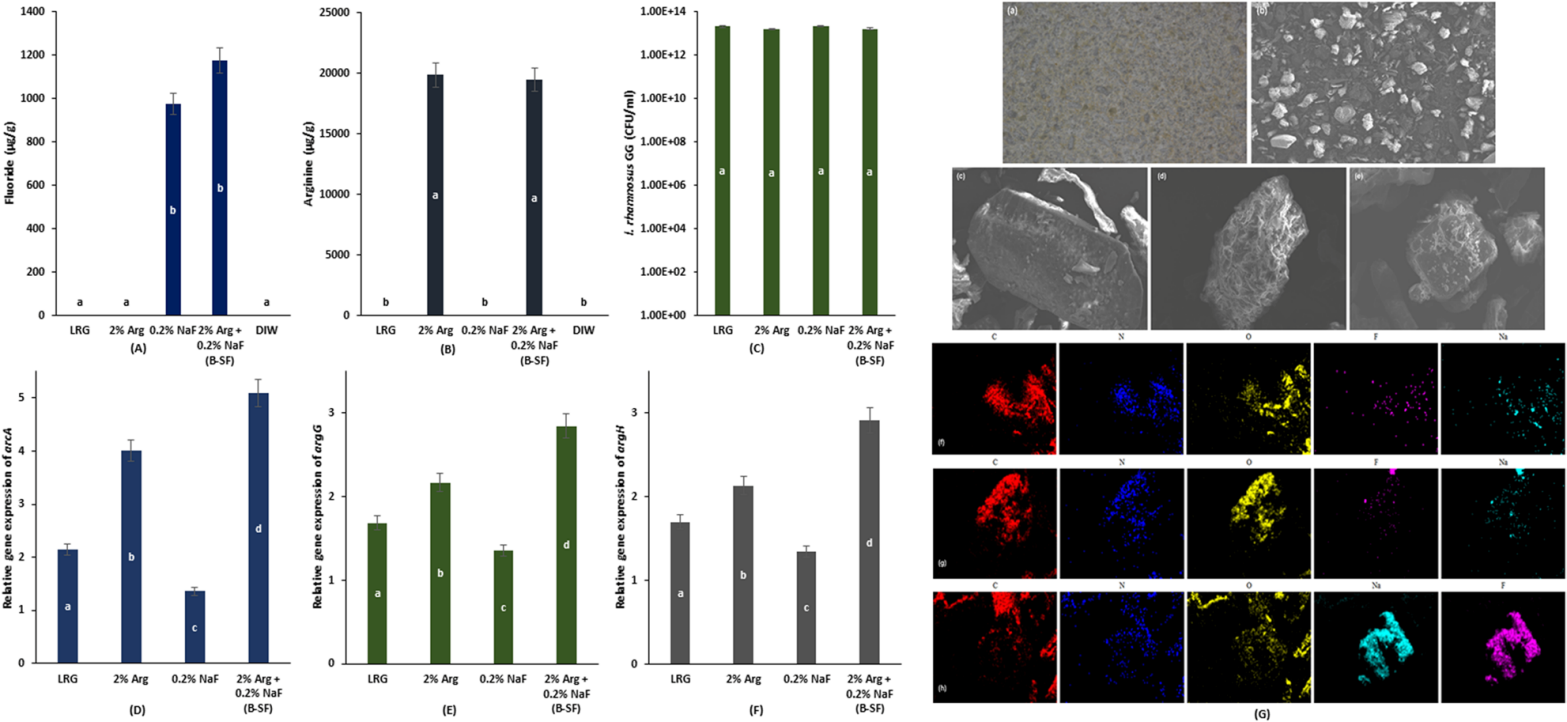
Bioavailability assessment of (**A**) F in B-SF therapy and respective controls; (**B**) Arg in B-SF therapy and respective controls; and (**C**) LRG in B-SF therapy and respective controls assessed by DNA isolation and quantitative real-time PCR. Relative expression of (**D**) *arcA *gene in B-SF therapy and respective controls; (**E**) *argG* gene in B-SF therapy and respective controls; and (**F**) *argH *gene in B-SF therapy and respective controls using RNA isolation and reverse transcriptase—real-time qPCR. (**G**) Macro-imaging, SEM imaging with EDX mapping of the proposed synbiotic-fluoride (B-SF) therapy: (**a**) 1:1 macro-image and; (**b**) SEM image (45 ×) of SF elixir. SEM image of (**c**) Arg; (**d**) probiotic supplement; and (**e**) NaF at 500 × from the SF elixir. SEM–EDX mapping of (**f**) Arg; (**g**) probiotic supplement; and (**h**) NaF from the SF elixir for C, N, O, F, Na elements. Different lowercase (a–d) English alphabets indicate significant differences between treatment groups at *p* < 0.05.

The F bioavailability in the B-SF and 0.2% NaF groups was significantly higher than 2% Arg, LRG, and DIW as almost negligible F content was found in the non-F groups (*p* < 0.05). No significant difference in F bioavailability was observed between B-SF and 0.2% NaF (*p* > 0.05) (Fig. 5A). Similarly, Arg detected in 2% Arg and B-SF was significantly higher than LRG, 0.2% NaF, and DIW as Arg was undetermined in non-Arg treated groups (*p* < 0.05) and no significant difference in Arg was discerned between 2% Arg and B-SF (*p* > 0.05) (Fig. 5B). Conversely, no significant difference in LRG bioavailability was observed among LRG, 2% Arg, 0.2% NaF, and B-SF groups (*p* > 0.05) (Fig. 5C). The results of B-SF therapy bioavailability showed that SF therapy did not affect the bioavailability of its basic components.

### Relative gene expression

The relative gene expression for *arcA*, *argG*, and *argH* gene of LRG was the highest in the B-SF group (*p* < 0.001). The least expression of these gene was observed in the 0.2% NaF treated LRG (*p* < 0.001). The gene expression in 2% Arg was significantly lower than B-SF, but higher than the LRG control (*p* < 0.05), whereby the LRG control demonstrated significantly higher gene expression than 0.2% NaF treated LRG (*p* < 0.05) (Fig. 5D–F).

The relative gene expression for* arcA *in the B-SF and 2% Arg treated LRG was 2.4 × and 1.9 × higher than that of the LRG control, respectively. For 0.2% NaF, the *arcA* expression was 0.6 × of the LRG control, demonstrating inhibited expression (Fig. 5D–F).

The relative gene expression for *argG* and *argH *in the B-SF and 2% Arg groups was 1.7 × and 1.3 × higher than that of the LRG control, respectively. Similar to *arcA*, the gene expression in the 0.2% NaF group was significantly inhibited, at 0.8 × of the LRG control (Fig. 5D–F).

Thus, the relative gene expression of *arcA*, *argG*, and *argH *was significantly increased with B-SF therapy.

### Synbiotic-Fluoride elixir imaging

The macro-imaged SF elixir at 1:1 magnification is shown in Fig. 5G(a) with simultaneously scanned SEM image at 45 × in Fig. 5G(b). The photographic and baseline scanned SEM image exhibits distribution of the probiotic supplement with Arg and NaF molecule. Figure 5G(c–e) shows SEM image of Arg, probiotic supplement, and NaF, respectively. Similarly, Fig. 5G(f–h) shows the SEM–EDX mapping for the individual components of SF admixture i.e. Arg, probiotic supplement, and NaF, respectively. Figure 5G(f) distinguishes a higher concentration of C, N, O as evident in the molecular formula to the amino acid—Arg; whereas the probiotic supplement (Fig. 5G(g)) displays higher proportional concentration of C and O indicating the presence of carbohydrates enmeshed probiotic supplement as available with Culturelle. The NaF compound is densely mapped for the Na and F elements (Fig. 5G(h)) whereby C and O can be differentiated in the background as possibly from the source of probiotic supplement.

Hence, the imaging results showed the unique presence of each component (prebiotic, probiotic, and NaF) in the SF commixture distinctly, indicating non-interacting molecules in its originally dispensable form and thus, conform to the result of F/Arg/LRG bioavailability.

## Discussion

The present study comprehensively evaluated and identified a potential SF therapy aimed at restoring ecological homeostasis. The study results show that 2% Arg with 0.2% NaF can enhance the growth of LRG. Thus, the null hypothesis that Arg combined NaF has no effect on the growth of LRG is rejected. The present study illustrates the possibility of using an integrated biotic approach that concomitantly include prebiotics, probiotics, and fluorides with profound synergism for maintaining ecological homeostasis without affecting their bioavailability.

Results of the CMA depicted that in the presence of 1%/2% Arg, the higher (> 4000 ppm) and lower (< 500 ppm) concentrations of NaF demonstrated indifference to LRG growth. It can be estimated that the high concentrations of NaF substantiated the antimicrobial effect against the planktonic cells by masking the effect of Arg on LRG as seen with *S. mutans*^39,40^. Although lower concentrations showed indifference to LRG growth, the ƩFIC > 1.5 distinctly showed a lower antimicrobial effect as compared to the higher concentrations (ƩFIC < 1.0). However, the discerned indifferent ƩFIC for lower NaF concentration with 1%/2% Arg can be explained on the relative expression of the cell growth by the combined intervention respective to the intervention alone. The lower concentrations of NaF alone demonstrated higher cell growth than the other higher concentrations; however, the relative effect of the combined lower NaF concentrations and 1%/2% Arg reduced the overall growth of LRG compared to the 0.05–0.4% NaF and 1%/2% Arg combinations. In addition, the indifference factor is reassuring as lower F concentrations have little or no effect on the biofilms of pathogens like *S. mutans* at concentrations ≤ 1200-ppm F^41,42^.

In contrast to the XTT assay results, the WST-8 assay revealed that LRG sustainability was improved by A-SF/B-SF while the metabolic activity of LRG was reduced when treated with 1% Arg + 0.4% NaF. Results of the probiotic cell metabolic assay should be interpreted with caution when relied on a single tetrazolium salt-based assay for deriving the conclusions. The WST-8 assay has higher sensitivity for cell and bacterial cultures as compared to the lower generation cell proliferation assays based on tetrazolium salts^43,44^. Therefore, both XTT and WST-8 assays were included in the present study.

The assay sensitivity results revealed that NaF concentration at 4000-ppm with 1% Arg inhibited the growth of LRG. Due to the presence of Arg in the combination, NaF at a concentration ≥ 4000-ppm demonstrated a bacterial inhibitory but not a bactericidal effect on the LRG planktonic cells. The results of the WST-8 metabolic assay was in concordance with the CMA results, which showed that the LRG growth inhibition was increased by 2-folds with NaF concentrations > 2000-ppm. However, similar growth inhibition was not detected with application of 1% Arg + 0.4% NaF. This could be due to the limitation of the CMA method, as the optical density values which indicated bacterial growth, is subjective to culture turbidity.

The LRG viability, biofilm thickness, cell density, vitality, and bacterial weight were enhanced when treated with A-SF/B-SF or 1%/2% Arg as compared to the LRG control, which is in agreement with the results of WST-8 assay. The SEM and 2D/3D confocal imaging results clearly identified well-grown LRG biofilm in the A-SF, B-SF, 1% Arg and 2% Arg groups, showing that Arg can enhance the growth of LRG through the multiple amino acid biosynthesis pathways of LRG that accommodate Arg metabolism^45^.

Furthermore, the biochemical parameters in the formed LRG biofilms were influenced by the combined effects of NaF and Arg on LRG. The F content in the biofilm treated with A-SF/B-SF was significantly reduced as compared to NaF alone. This may be due to the interaction of NaF with Arg, leading to the formation of Arg-F complex, hindering the availability of the free F ions in the biofilm^46^. Similarly, the amine concentration was significantly reduced in the A-SF/B-SF-treated LRG biofilm, affirming the interaction of amino acid with negatively charged F ions. As an advantage to the A-SF/B-SF treatment, the lactic acid generated in the spent media was significantly reduced when compared to the Arg (1%/2%) and LRG control. The A-SF/B-SF applications increased the activity of amino-acid biosynthesis pathways of LRG, consequently suppressing the lactic acid generating activity of lactobacilli.

Another reason is that the low molecular weight lactic acid producing potential of LRG is active at low pH^47^. With supplementation of Arg, the pH of the growth conditions increases and thus, the lactic acid production activity of LRG is reduced. The reduced activity in the A-SF/B-SF groups in this study may be due to the longer intra-cellular retention of Arg-F complexes, whereby the metabolism of amino acid was delayed, thereby maintaining high pH conditions non-conducive to LRG lactic acid production. The rationale for the estimated biochemical parameters with SF therapy is further affirmed through pH evaluation of the spent media. Although in this study, the difference between baseline and 24 h pH was significant, pH of the spent media was little affected by A-SF, B-SF, and 2% Arg treated LRG after 24 h, which revealed that a high pH condition (> 7.0) was continuously maintained and might have reduced the lactic acid producing potential of LRG treated with A-SF/B-SF.

The concept that LRG has a good pH buffering capacity^48^ explains the study finding that the buffer capacity for LRG after 24 h of A-SF therapy and 0.2% NaF treated LRG were similar to LRG but failed to maintain a pH close to that of the B-SF therapy. The B-SF therapy lead to a higher buffer capacity (although lower than 2% Arg) because of the 2 × supplementation of Arg as the pH was affected by the Arg nativity (pKa = 12.38).

The reduced buffer capacity of B-SF compared to 2% Arg can be further explained based on the evident pH changes, whereby even the baseline pH of the combined Arg and NaF was lower than Arg alone. The interaction of Arg with NaF is a potential reason for such an effect, which the sequel of Arg-F complex reduced the pKa of Arg. The results of the buffer capacity of the added buffers (NaF/Arg/NaF + Arg) and qualitatively identified biofilm pH distinctly presented that B-SF therapy maintained the pH close to neutral, even after 24 h. The resistance of B-SF therapy to pH change is beneficial for its application in harsh environments (with lower pH) like oral cavity.

Based on the results discussed above, B-SF therapy was chosen to further explore its mechanistic properties; hence, fluoride/Arg/LRG bioavailability, gene expression, and SF elixir imaging were evaluated in this study. The bioavailability of the B-SF therapy was assessed in the DIW as the composition can be easily delivered in the form of mouth rinse, or a pharmaceutical supplement intended for ingestion. The bioavailability of the individual components of the B-SF therapy compared to the respective controls remained unaffected indicating no interaction. Similarly, the B-SF therapy imaging showed that each component (Arg, NaF, LRG) was uniquely present in the commixture, although subjected to homogenization. The rationale for unaltered bioavailability is the environment, nourishment, and duration for metabolism of the prebiotic-F in the presence of probiotics. The present study examined the effect of Arg and NaF on LRG after 24 h (duration) when both the environment (anaerobic) and nourishment (MRS broth) were favourable for the growth of LRG. Having no interactions in the B-SF therapy at a primary dispensable stage may not occur under other conditions.

In this study, in the presence of F, Arg associated ADS (*arcA*) activity was increased more than 2-folds compared to the control; whereas, the *argG* and *argH* gene expression was increased by 1.5-folds. Similarly, the expression for *arcA*, *argG*, and *argH* with Arg alone was significantly higher than the control but lower than B-SF therapy. The increased ADS activity can be attributed to the fundamental metabolism process of Arg, whereby Arg is metabolized to ornithine, citrulline, CO_2_, and ATP by several oral commensals termed as arginolytic bacteria^49^. Although LRG primarily metabolizes Arg, it sequentially adjoins the next stage in the metabolism process which is evident by the *argG* and *argH* expressions and thus, cannot be regarded as an absolute arginolytic bacteria. Furthermore, the presence of F exerts an electronegative force on the guanidinium group of Arg, generating strain on Arg which limits its availability, leading to prolonged metabolism as seen in the B-SF treatment on LRG in this study showing an intensified gene expression.

**Figure 6:**
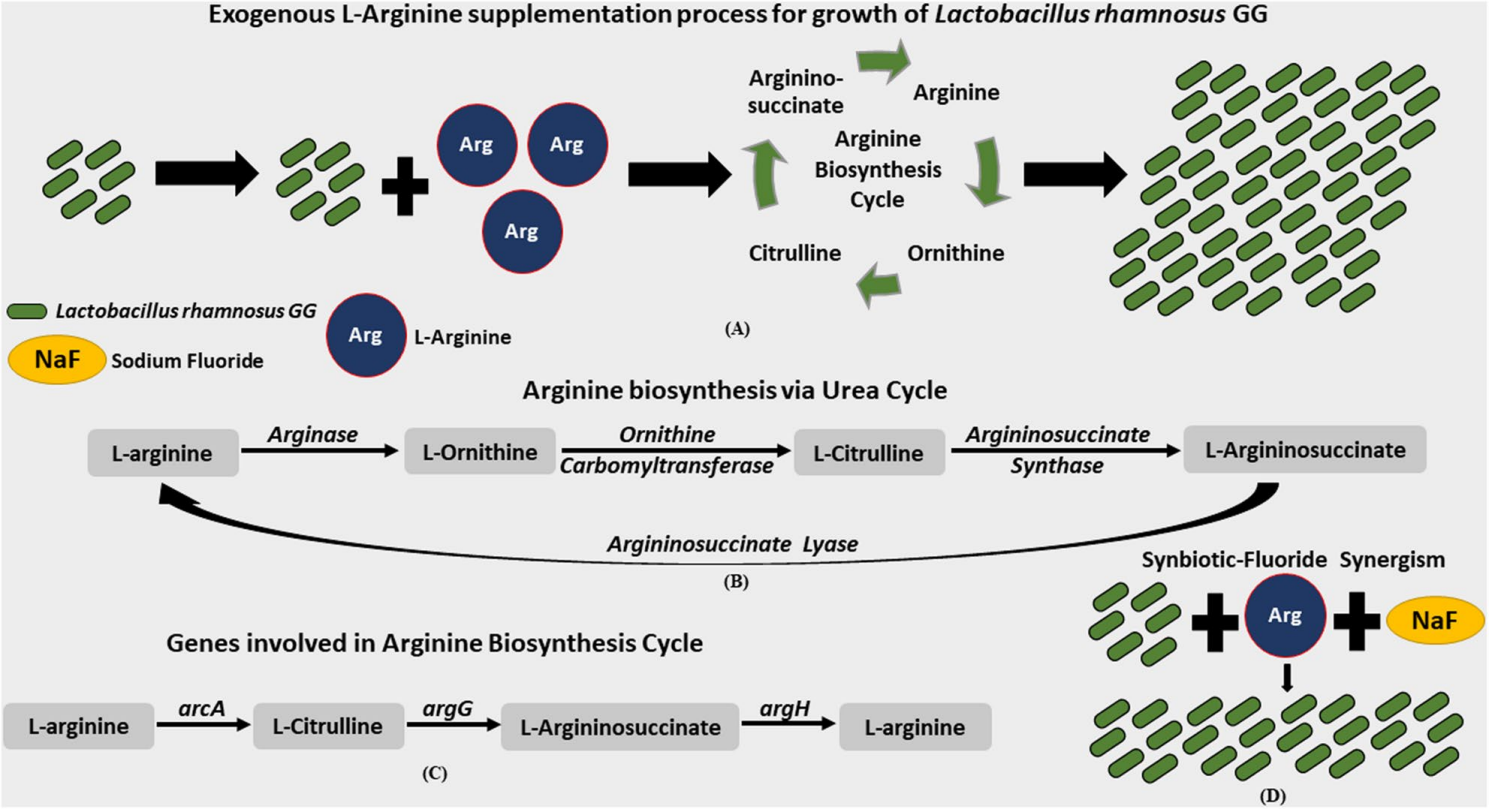
Mechanism of action of the proposed SF therapy. (**A**) Exogenous Arg supplementation for growth of LRG. (**B**) Arginine biosynthesis via Urea Cycle. (**C**) Genes involved in Arg Biosynthesis Cycle. (**D**) Effect of SF synergism for growth of LRG.

Finally, we present the mechanism of action of the proposed SF therapy in Fig. 6. When LRG is co-delivered with Arg as synbiotics, the amino acid enters the arginine biosynthesis cycle of the LRG. *Lactobacillus rhamnosus* GG has limited potential to synthesize amino acids for its growth and Arg acts as a source of exogenous amino acids^50,51^. The amino acid (Arg) is further converted to ornithine, citrulline, and arginino-succinate prior to being recycled as Arg (Fig. 6A). The cycle continues and supports the growth of LRG. Therefore, compared to LRG alone, combination of LRG with Arg has a significantly higher growth and thus can be developed as a synbiotic therapy.

Furthermore, Arg biosynthesis via urea cycle is a step-wise process as shown in Fig. 6B. The supplemented Arg is converted to L-ornithine by a manganese-containing enzyme—arginase. L-ornithine is converted to L-citrulline in the presence of ornithine carbomyltransferase by catalysing the reaction between L-ornithine and carbomyl phosphate. The formed citrulline is further catalysed by argininosuccinate synthase to synthesize L-arginino-succinate, which is catalysed by argininosuccinate lyase to Arg. Thus, the supplementation of Arg regulates the growth of LRG via the urea cycle.

Figure 6C shows the role of LRG genes in the LRG growth by Arg supplementation, which is evident by the results of the present study. An ADS positive *arcA* gene is responsible for conversion of Arg to L-citrulline; whereas, the *argG* and *argH* genes convert L-citrulline to L-argininosuccinate and L-argininosuccinate to Arg, respectively. As per the results of the present study, it is perceivable that Arg with NaF demonstrated increased influence on the relative gene expression as compared to Arg alone. The interactive potential of the positively charged guanidinium group of Arg and the electronegative F prospectively prolonged Arg metabolism in the Arg biosynthesis. The uphold function of Arg-F complex aids in the sustenance of LRG and stimulates the pathway for the bacterial cell growth (Fig. 6D). Therefore, in this study, the combination of 2% Arg (prebiotic), 0.2% NaF, and LRG (probiotic) together demonstrated real-time translation of the integrated biotic treatment targeted for restoring ecological homeostasis by the evident action of LRG and its concurrent functional elicitation by prebiotic-F interaction.

The present paper demonstrates that probiotic formulation enhancement can be substantiated through synergistic synbiotic means. This will further augment the immunological functions, protect against pathogenic bacteria, and aid in replenishment of the microflora. *Lactobacillus rhamnosus *GG modulates cariogenic microbiome associated dysbiosis and improves periodontal health and halitosis^52^. The probiotic LRG inhibits the growth of *P. gingivalis, *so the potential threat of Arg promoting the overgrowth of oral anaerobes (*P. gingivalis) *can be countered^24,53^. Apart, the possible halitosis caused by ammonia generation from Arg may be alleviated. This is because LRG has a potential to inhibit volatile sulphur compounds (VSC) production by VSC producing bacteria as certain lactobacilli species probiotics have been shown to reduce oral malodour^54,55^. Hence, the combination of Arg, NaF and LRG (in the proposed proportion) seems beneficial with potential applications for the control of dental caries, periodontal diseases and halitosis in the oral cavity.

The present study focuses on the mechanistic properties of Arg and NaF affecting the growth of LRG, while projecting the scope of the combined SF therapy for the applications in oral cavity. However, the effect of the SF therapy on other oral pathogens (e.g. *S. mutans*) needs further evaluation. Before making definitive recommendations for general use, further studies on the interactive effect of other commensals and pathogens with the SF treatment are required to provide a broader understanding of the precise dynamics of the proposed therapy. Notwithstanding the above, clinical use of the SF approach is safe as each component of the therapy has been tested for use in humans. The SF therapy presents a potent drug synergism with a potential for wide applications and anticipated beneficial effects.

## Methods

### Probiotic, prebiotic, synbiotic, fluoride

*Lactobacillus rhamnosus* GG was isolated from a probiotic nutritional supplement—Culturelle (i-Health, Inc., Denmark) known to restore the natural balance of pro-healthy bacteria in the digestive tract. The probiotic supplement is claimed to support the natural immune defences for overall health and well-being. The dietary supplement was cultured in MRS broth at 37 °C for 72 h under anaerobic conditions (85% N_2_, 10% H_2_, 5% CO_2_). The cells were then adjusted to a concentration of 10^7^ cells/ml in MRS broth (pH: 7.00) with concentration estimated using UV–Vis Spectrophotometer (Beckman Coulter, CA, USA) at OD_600_ nm by Mc Farland spectrophotometric method. The strain was further matched to an in house ATCC strain.

*Lactobacillus rhamnosus* ATCC 53103 by DNA isolation and real-time quantitative polymerase chain reaction (qPCR), which was thereby included for standard curve calibration and DNA quantification by qPCR.

L-arginine (a prebiotic) at concentrations 0.25–2% *by wt.* serially diluted in sterilized 0.9% NaCl were used in the study, while the vehicle (0.9% NaCl) was treated as a control. The synbiotic investigated in the present study, combined Arg and LRG, was developed based on the results of our previous study which showed that L-arginine could enhance the growth of LRG^56^. The F source used in the study was NaF at concentrations 15,625–32,000 ppm serially diluted in sterilized 0.9% NaCl, and the vehicle (0.9% NaCl) was used as a control. Therefore, the SF therapy addressed in the study was the combination of LRG, Arg, and NaF.

### Checkerboard microdilution assay (CMA)

**Figure 7:**
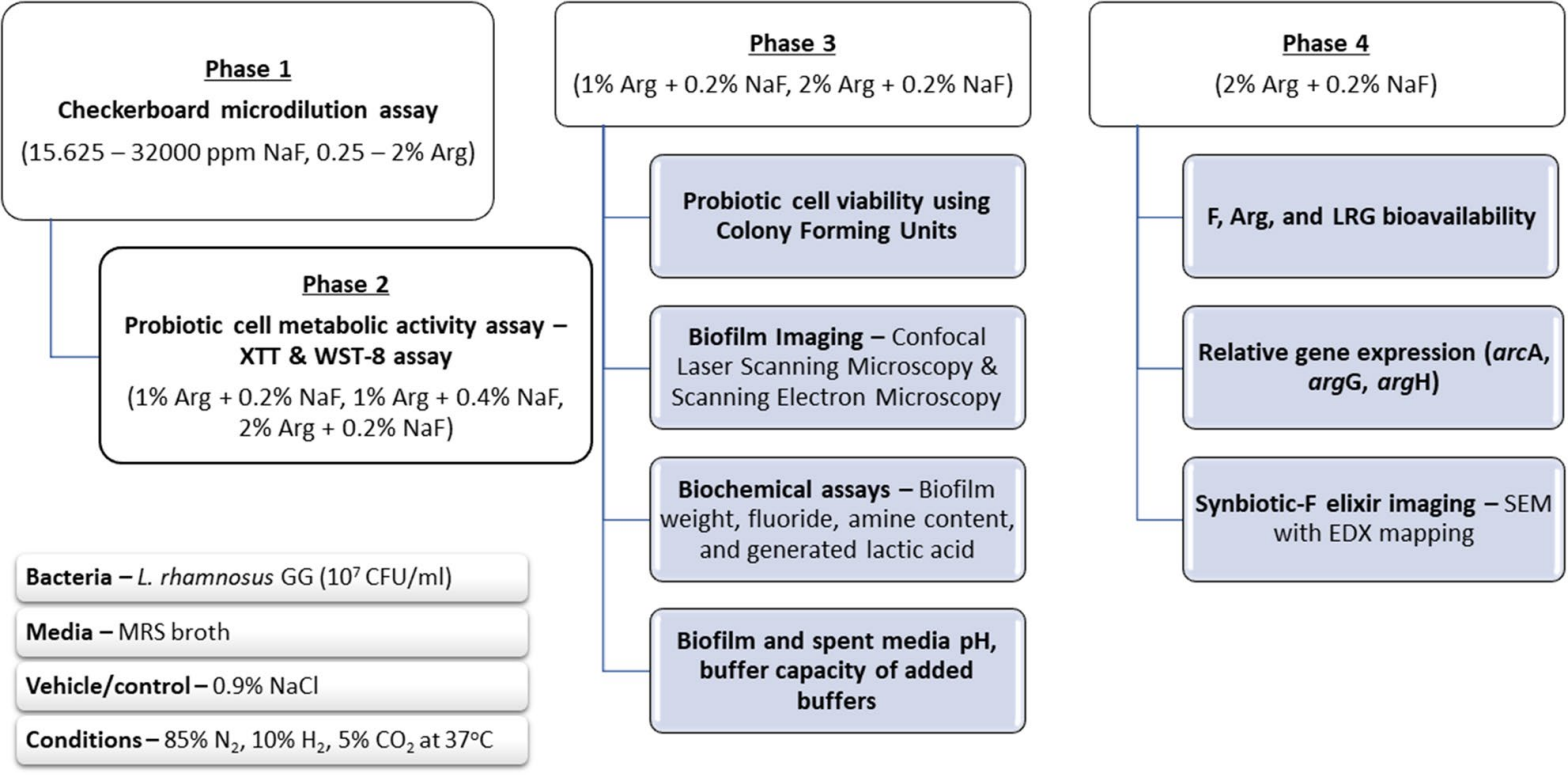
Experimental study design.

The synergistic/antagonistic effect of the combined NaF and Arg on the growth of planktonic LRG was determined using CMA subjected to the assessment of the controls. The assay was performed in a 96-well microplate which contained LRG, NaF, Arg, the vehicle control or the medium in equal volume. After 24 h incubation in an anaerobic chamber (85% N_2_, 10% H_2_, 5% CO_2_; 37 °C), the growth of LRG was examined by spectrophotometer (Beckman Coulter, CA, USA) at OD_600_ nm. The minimum inhibitory concentration (MIC) was outlined for each test agent for calculation of fractional inhibitory concentration (FIC). The FIC was the ratio of the MIC of the test agents used in combination to the MIC of the test agents used alone^57^. The summation of the individual FICs (ƩFIC) i.e. FIC Index were computed to analyse synergism/antagonism. A ƩFIC < 0.5 indicated drug synergism, 0.5 to 4.0 was regarded as indifference, while ƩFIC > 4.0 demonstrated antagonism^24^. The results of the CMA were used to identify potential SF combinations for further experimental evaluation using the study design for CMA whereby the experiments were performed in a 96-well microplate which contained the probiotic LRG with/without NaF/Arg. As a standard experimental study design (Fig. 7), following the CMA, for all further experiments the biofilms were grown for 24 h under the anaerobic conditions (85% N_2_, 10% H_2_, 5% CO_2_; 37 °C).

### Probiotic cell metabolic activity

To further confirm the outlined evaluations by CMA, XTT and WST-8 assay were undertaken to analyse LRG metabolic activity representative of the LRG growth for 1% Arg + 0.2% NaF, 1% Arg + 0.4% NaF, and 2% Arg + 0.2% NaF.

For XTT assay, the reagent was prepared with XTT developer reagent and electron mediator solution supplied with the XTT assay kit (abcam—ab232856, USA). After 24 h incubation as with CMA, the planktonic cells grown to biofilms were washed with PBS prior to receiving the reagents. The reagent was subjected to tilt-mix on an orbital shaker for 5 min under sealed conditions to avoid light interaction with the reagent. Then, the biofilms treated with the reagents were incubated in a CO_2_ incubator at 37 °C for 4 h. After 4 h, the suspensions were centrifuged at 3,000 rpm for 5 min and the aliquot of the supernatant was used to measure absorbance at OD_450_ nm^58^.

The WST-8 assay was performed as per our previous study^59^. Briefly, the reagent was prepared as per manufacturer’s instructions on the assay kit (Dojindo Laboratories, Japan). As per XTT, the biofilms were washed with PBS prior to receiving the reagents. Then, the biofilms were subjected to the reagents and incubated for 4 h in a CO_2_ incubator at 37 °C. After incubation, the plate was centrifuged and the supernatant was measured for absorbance at OD_450_ nm. All OD_450_ nm readings with XTT and WST-8 assay were normalized to the irrespective controls.

### Probiotic cell viability

The LRG viable cells at 24 h after incubation in an anaerobic chamber (85% N_2_, 10% H_2_, 5% CO_2_; 37 °C) were determined by manually counting colony forming units (CFU) on petri-dishes with Horse Blood Agar (HBA). Prior to preparing the biofilm suspension, the biofilms were washed with PBS. The suspensions of the biofilms were prepared using the MRS broth. The suspensions were spiral plated (Autoplate 4000, Spiral Biotech Inc., MA, USA) on the HBA containing dishes and incubated in an anaerobic chamber (85% N_2_, 10% H_2_, 5% CO_2_; 37 °C) for 72 h prior to counting the CFUs. The counted CFUs were estimated based on the diluted suspensions with final derived measures in CFU/ml adjusted for dilutions.

### Confocal laser scanning microscopy

To characterize the biofilms by confocal imaging, the biofilms were grown on hydroxyapatite discs (5 mm × 2 mm; Clarkson Chromatography Products, Inc, USA) with the test agents. After washing the 24 h biofilms with PBS, the biofilms were stained with SYTO 9 and Propidium Iodide (LIVE/DEAD *BacLight* Bacterial Viability Kit). The stained biofilms were scanned with confocal laser scanning microscope (Olympus, FLUOVIEW FV1000, USA) as per manufacturer instructions. The scanning was performed at 3 randomly selected biofilm fields at 100 × magnification. The scanned images were stacked to obtain a 3-D scaled biofilm image which exhibited the biofilm thickness with FV10-ASW ver. 4.2b (Olympus Corporation, Shinjuku, Tokyo, Japan). The biofilm thickness was quantified in µm for test groups and control for further statistical analysis. The 2-D biofilm images qualitatively informed vital growth of LRG subjected to the test agents and alongside controls.

### Scanning electron microscopy

The biofilms for confocal imaging were further subjected to imaging by scanning electron microscopy (SEM) as per our previous study^59^. The SEM was performed to assess the surface topography of the treated biofilms. Briefly, the biofilms were fixed overnight with 2.5% glutaraldehyde and serially dehydrated with the graded series of ethanol at concentrations 70%—85%—95% and then absolute ethanol at every 30 min. The biofilms were air dried at room temperature in a dry box overnight prior to sputtering (MSP-2S, IXRF systems, USA) with platinum-palladium. The SEM unit (SU1510, Hitachi, Japan) was set at 15 kV, 8000 × to capture the biofilm images at 3 randomly selected view fields in the biofilm within the confines of the discs.

### Bacterial weight, biofilm fluoride and amine content, generated lactic acid

To determine bacterial weight in the biofilm and biofilm F/amine content, after washing with PBS the 24 h biofilms were re-suspended in MRS broth to do the assays. The variables were estimated using a protocol adopted in a previous study^60^. Eppendorf tubes containing biofilm samples were centrifuged at 10,000 rpm for 5 min. The media was completely discarded and the bacterial pellet weight was determined. The bacterial pellets were extracted with 1 mol/L HCl for 1 h at room temperature. The extracted pellets were further neutralized with equal volume of 1 mol/L KOH. The neutralized samples were further buffered with TISAB II before determining biofilm F content with the calibrated fluoride ion-selective electrode (F-ISE) (R^2^ > 0.99).

To estimate the biofilm amine content indicating the concentration of amino acids, Arg standards were used and amine content was detected by o-pthaldialdehyde derivatization method. A working solution of o-phthaldialdehyde dissolved in absolute ethanol, β-mercaptoethanol, Na_2_CO_3_, and deionized water (DIW) was prepared. The neutralized samples were suspended in the working solution (1:10) in a 96-well microplate for fluorescence assays. Eight Arg standards were prepared in 2-folds dilution to construct a calibration curve for deriving µg/g of amine content. The plate was read spectrophotometrically using end-point fluorescence with excitation at OD_340_ nm and emission at OD_455_ nm.

The generated lactic acid was estimated in the spent media. The spent media was sampled and centrifuged at 10,000 rpm for 5 min. The supernatant aliquot was used to determine the lactic acid. The aliquot was suspended in 0.2% FeCl_2_ solution and vortexed for 60 s. A series of standard solutions of lactic acid were prepared to construct a calibration curve based on the absorbance measured spectrophotometrically. The absorbance of test solutions and standards was measured at OD_390_ nm with determination of lactic acid based on the calibration curve. The amount of lactic acid was presented in g/L as per a previous study^61^.

### pH of spent media, biofilm, and buffer capacity of added buffers

The spent media pH was determined using a pH electrode calibrated to 4.01, 7.00, and 10.01 pH standards. The pH electrode attached to a potentiometer (Orion 2700, Oakton Instruments, USA) was stationed on a magnetic stirrer to enable continuous stirring with a micro-magnetic bar of the spent media during measurements. The pH of the media after subjecting it to treatments was measured as baseline and further pH measurements were made after 24 h incubation in an anaerobic chamber.

After 24 h, the spent media was removed and the biofilm was qualitatively assessed using pH colorimetric indicator assay against a set of standards. Five HEPES buffer standards (50 mmol/L) of pH: 4.50, 5.50, 6.50, 7.50, and 8.50 were prepared and subjected to pH colorimetric indicator (Hydrion Buffer Color Key Preservative, USA). Similarly, the 24 h biofilms were treated with the pH indicator and then the microplate was scanned to estimate the pH change with different treatments.

The buffer capacity of the added buffers (NaF and Arg) were computed based on a previous study^62^ using the following formula –

$$B = 2.303 \left[ {\frac{{Ka \left[ {buff} \right]\left[ H \right]}}{{\left( {Ka + \left[ H \right]} \right)^{2} }} + \left[ H \right] + \frac{Kw}{{\left[ H \right]}}} \right]$$

whereby; *B* = buffer capacity of the added Arg/NaF, *Ka* = dissociation constant for the added Arg/NaF, [*buff*] = concentration of the added arginine Arg/NaF/Arg + NaF. Kw = dissociation constant for water, [H] = hydrogen ion concentration of slurries with added Arg/NaF/Arg + NaF.

### Bioavailability of synbiotic-fluoride therapy

The identified SF therapy was further examined for bioavailability of F, Arg, and LRG. The combined F, Arg, and LRG (from the probiotic nutritional supplement) were suspended in 10 ml of DIW and thoroughly vortexed for 60 s to obtain homogeneous suspension. The bioavailability with each component was estimated as per the following method.

#### Fluoride

The F content was determined similar to the procedure mentioned above using F-ISE. Aliquot of the suspension was drawn and buffered using TISAB II. The measurements were made against external NaF standards of 0.1, 1, 10, 100, 1000 µg/g F concentration which were similarly buffered with TISAB II as per the suspended SF therapy in DIW. The constructed calibration plot was monitored for stability before, during, and after the experiment. During determination of F concentrations, the samples were continuously stirred on a magnetic stirrer at 250 rpm using micro-magnetic bars.

#### Arginine

Arginine in the suspensions was determined spectrophotometrically by o-pthaldialdehyde derivatization method similar to the amine detection method. Arginine standards were prepared from 10 µg/g concentrations. Therefore, the suspended samples were diluted 4000 times to match the range of the standard curve and adjusted for dilution after characterization. The suspensions and the o-pthaldialdehyde containing working solutions were introduced in the 96-well plate for fluorescence assay and the end-point fluorescence was measured with excitation at OD_340_ nm and emission at OD_455_ nm.

#### DNA quantification with real-time quantitative polymerase chain reaction

The DNA quantification using real-time qPCR was performed as per our previous study^59^. Aliquot of SF therapy suspension was used for DNA isolation. The suspension was centrifuged at 14,000 rpm for 10 min and the supernatant was discarded. The sediment pellet was suspended in 20 mM Tris–HCl, pH: 7.00; 2 mM EDTA; 1.2% Triton buffer and the bacterial cells were lysed with 20 mg/ml lysozyme by incubating in a water bath at 37 °C overnight. The DNA isolation was performed using QIAamp DNA Isolation kit (Qiagen, Hilden, Germany) following manufacturer instructions. *Lactobacillus rhamnosus *GG ATCC 53103 and the cultured *L. rhamnosus* GG from the dietary supplement were subjected to DNA isolation and served as positive controls for the real-time PCR reaction. The primers used in the present study are listed in Table 1. The Taqman probe (Applied Biosystems, USA) for LRG detection used in the present study was 5′-6FAM-CGGATTTCCAAAGCAATTCTTAACGATGAAAATG-TAMRA-3′^63^. The probes and F/R primers were mixed with Taqman universal PCR mix and isolated DNA for the PCR reaction in an optical 96-well PCR reaction plate against the reference standards. The cycle reaction conditions were set as: 50 °C/2 min; 95 °C/10 min; 50 cycles of 95 °C/15 s and 58 °C/1 min. The PCR reaction was performed using Step One Plus (Applied Biosystems, USA).

**Table 1:** Primers used for real-time PCR.

Target	Primer sequence
*arcA*	F: 5′—CGA CTT GAA CTT GGA CCA CAG A—3’ R: 5′—TCC AGC TTA AGC GCA TCC TT—3’
*argG*	F: 5′—CCC AAG CCG TCG TCA ATG—3’ R: 5′—GGG ACG GCA CTG CCT TT—3’
*argH*	F: 5′—GCA CCG CAA ATC CAA GAA GA—3’ R: 5′—GCG ATT GAC GGC TGC TTT T—3’
*L. rhamnosus* GG 16S rRNA	F: 5′—GGC GGC TGT CTG GTC TGT AA—3’ R: 5′—TCC TGT TCG CTA CCC ATG CT—3’
*L. rhamnosus* GG	F: 5′—CAGAAATCAAAGAAGACAAACTCGTTA—3’ R: 5′—CCATGTAAACGGACAATGGGAGT—3’

### Relative gene expression profile

The relative gene expression for *arcA*, *argG*, and *argH *genes was measured for the selected SF therapy against the respective controls similar to our previous study^59^. After 24 h growth of treated LRG, the biofilms were washed with PBS. The LRG biofilms were sampled in 0.9% NaCl and then centrifuged at 14,000 rpm for 10 min. The supernatant was discarded and the pellet was suspended in freshly-prepared TE buffer—10 mM Tris–HCl, 1 mM EDTA (pH: 7.00) containing 0.4 mg/ml lyzoyme and kept at room temperature for 15 min. The RNA isolation was done using the SV RNA isolation kit (Promega, USA) as per manufacturer instructions. Then, the isolated RNA was assessed for purity by measuring absorbance at A_260/280_ on a NanoDrop spectrophotometer (ThermoFisher Scientific, USA). Reverse transcription of RNA to cDNA was synthesized using Superscript Reverse Transcriptase (Invitrogen, USA) as per manufacturer instructions. The real-time PCR reaction performed in an optical 96-well PCR plate included the SYBR Green master mix, F/R primers, and cDNA. The primers for the genes used in the study are presented in Table 1. The reaction was performed using Step One Plus (Applied Biosystems, USA) with the cycle condition set as 50 °C/2 min; 95 °C/10 min; 50 cycles of 95 °C/15 s and 58 °C/1 min. The relative gene expression was estimated after the cycle threshold values were normalized to the reference house-keeping 16S rRNA gene.

### Imaging of synbiotic-fluoride elixir

Prior to imaging, each component—prebiotic (Arg), probiotic supplement, and NaF were dispensed in the determined proportion for B-SF in a sterilin tube (Bijou, Thermo Scientific, Newport, UK). The proportional components were thoroughly homogenized in the mixture by subjecting the tube to a vortex for 60 s. The elixir was further drawn for macro- and SEM-imaging. The SEM imaging was supplemented with Energy Dispersive X-ray Spectroscopy (EDX) analysis that assisted in the identification of the individual components in SF elixir.

The SF elixir macro-imaging was done using 100-mm telephoto prime macro lens (Canon EF Macro USM, Japan) attached to a D-SLR camera (Canon 550D, Japan) adapted on a photographic stand whereby elixir was suspended in a 5.5 cm petri-dish on an image light box. The image was taken at 1:1 magnification set at manual focus.

Further, the elixir was suspended on a carbon tape attached to a stub and sputtered with platinum-palladium prior to stationing the stub in the SEM unit (SU1510, Hitachi, Japan). The SF admixture was scanned at baseline 45 × and identified for the elements—prebiotic (Arg), probiotic (LRG) supplement, and NaF. Each element in the commixture was discerned by SEM–EDX prior to imaging the scanned component at 500x. The SEM–EDX of the individual components of SF elixir was mapped for C, N, O, F, and Na elements.

### Statistical analysis

All experiments were performed at least in triplicates and at 3-separate time points. The data were analysed using SPSS v. 25 (IBM Statistics Inc., USA). The data for XTT and WST-8 assay were analysed using Kruskal–Wallis 1-way ANOVA followed by Dunn-Bonferroni’s post-hoc test. The data for probiotic cell viability to determine CFU was log transformed prior to further statistical analysis. The data for log transformed probiotic cell viability, biofilm thickness, bacterial weight, biofilm F/amine content, generated lactic acid, measured pH and derived buffer capacity of the added buffers, F/Arg/LRG bioavailability, and relative gene expression were analysed using 1-way ANOVA with Tukey’s HSD post-hoc test. The difference between the baseline pH and the pH measured after 24 h of the spent media was assessed by paired t-test. The statistical significance level for all tests was set at *p* < 0.05.

## Conclusion

Based on the results of this series of investigations, it is concluded that

1. Combination of L-arginine (1%/2%) with NaF (2000-ppm) can enhance the growth of *Lactobacillus rhamnosus* GG.
2. Combining 2% L-arginine and 2000-ppm NaF with *Lactobacillus rhamnosus* GG provides optimum synbiotic-fluoride synergism.
